# Multidisciplinary team approach in acute myocardial infarction patients undergoing veno-arterial extracorporeal membrane oxygenation

**DOI:** 10.1186/s13613-020-00701-8

**Published:** 2020-06-16

**Authors:** David Hong, Ki Hong Choi, Yang Hyun Cho, Su Hyun Cho, So Jin Park, Darae Kim, Taek Kyu Park, Joo Myung Lee, Young Bin Song, Jin-Oh Choi, Joo-Yong Hahn, Seung-Hyuk Choi, Jin-Ho Choi, Kiick Sung, Hyeon-Cheol Gwon, Eun-Seok Jeon, Jeong Hoon Yang

**Affiliations:** 1 Division of Cardiology, Department of Internal Medicine, Heart Vascular Stroke Institute, Samsung Medical Center, Sungkyunkwan University School of Medicine, 81 Irwon-ro, Gangnam-gu, Seoul, 06351 Republic of Korea; 2 Department of Thoracic and Cardiovascular Surgery, Samsung Medical Center, Sungkyunkwan University School of Medicine, Seoul, Republic of Korea; 3 Department of Pharmaceutical Services, Samsung Medical Center, Seoul, Republic of Korea; 4 Department of Critical Care Medicine, Samsung Medical Center, Sungkyunkwan University School of Medicine, Seoul, Republic of Korea

**Keywords:** Extracorporeal membrane oxygenation, Multidisciplinary team, Acute myocardial infarction, Cardiogenic shock

## Abstract

**Background:**

Limited data are available on the impact of a specialized extracorporeal membrane oxygenation (ECMO) team on clinical outcomes in patients with acute myocardial infarction (AMI) complicated by cardiogenic shock (CS). This study evaluated whether specialized ECMO team is associated with improved in-hospital mortality in AMI patients undergoing veno-arterial (VA) ECMO.

**Methods:**

A total of 255 AMI patients who underwent VA-ECMO were included. In January 2014, a multidisciplinary ECMO team was founded at our institution. Eligible patients were classified into a pre-ECMO team group (*n* = 131) and a post-ECMO team group (*n* = 124). The primary outcome was in-hospital mortality.

**Results:**

In-hospital mortality (pre-ECMO team vs. post-ECMO team, 54.2% vs. 33.9%; *p* = 0.002) and cardiac intensive care unit mortality (pre-ECMO team vs. post-ECMO team, 51.9% vs. 30.6%; *p* = 0.001) were significantly lower after the implementation of a multidisciplinary ECMO team. On multivariable logistic regression model, implementation of the multidisciplinary ECMO team was associated with reduction of in-hospital mortality [odds ratio: 0.37, 95% confidence interval (CI) 0.20–0.67; *p* = 0.001]. Incidence of all-cause mortality [58.3% vs. 35.2%; hazard ratio (HR): 0.49, 95% CI 0.34–0.72; *p* < 0.001) and readmission due to heart failure (28.2% vs. 6.4%; HR: 0.21, 95% CI 0.08–0.58; *p* = 0.003) at 6 months of follow-up were also significantly lower in the post-ECMO team group than in the pre-ECMO team group.

**Conclusions:**

Implementation of a multidisciplinary ECMO team was associated with improved clinical outcomes in AMI patients complicated by CS. Our data support that a specialized ECMO team is indispensable for improving outcomes in patients with AMI complicated by CS.

## Background

Cardiogenic shock (CS) is the main cause of mortality in patients with acute myocardial infarction (AMI) [1, 2]. Despite advancements in reperfusion and pharmacological therapy, the short-term mortality rate of patients with AMI complicated by CS remains unacceptably high [1, 2]. Particularly, in refractory CS not responding to conventional medical therapies, in-hospital mortality rate reaches 50% to 60% [3, 4] and mechanical support such as veno-arterial (VA) extracorporeal membrane oxygenation (ECMO) is recommended in both the latest American Heart Association and the European Society of Cardiology guidelines (classes IIA and IIB, respectively) [5, 6].

These poor outcomes are due to complex and hemodynamically diverse state of cardiogenic shock [7, 8]. The high-acuity of maintaining ECMO and the interaction between native heart and VA-ECMO may also be related to the poor outcomes [9, 10]. In particular, running VA-ECMO is associated with many serious complications, which may contribute to further increase in morbidity and mortality [9, 11–13]. Accordingly, related organizations recommended that these patients be managed by a collaborative multidisciplinary team with trained specialists [8, 14]. However, for AMI complicated by CS, which is the most common cause for the use of VA-ECMO [15], the impact of a multidisciplinary approach on the clinical outcome has not been investigated.

Therefore, we sought to identify whether a multidisciplinary ECMO team is associated with improvements in in-hospital mortality among patients with AMI complicated by CS who underwent VA-ECMO.

## Methods

### Study population

**Fig. 1:**
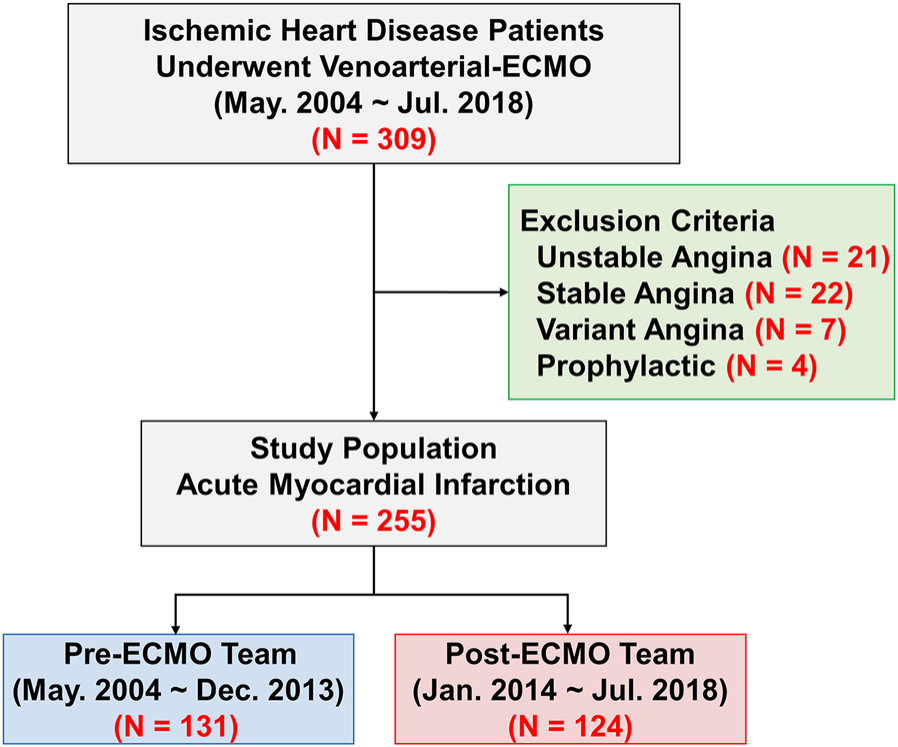
Study flow. *ECMO* extracorporeal membrane oxygenation

The study population was derived from the prospective institutional VA-ECMO registry of Samsung Medical Center in Seoul, Republic of Korea from May 2004 to July 2018 (Fig. 1). From this registry, AMI patients complicated by CS were included in the analysis. AMI was defined as evidence of myocardial injury (defined as an elevation of cardiac troponin values, with at least one value above the 99th-percentile upper-reference limit) with necrosis in a clinical setting, consistent with myocardial ischemia [6]. CS was defined as persistent hypotension (systolic blood pressure < 90 mmHg) for 30 min or a state that required inotrope or vasopressor support to achieve a systolic blood pressure of more than 90 mmHg despite adequate filling status, with signs of hypoperfusion [6]. VA-ECMO was applied to patients with medically refractory CS that did not respond to inotropes and vasopressors, or cardiac arrest that was not resuscitated with advanced cardiac life support [3, 9]. Patients who received VA-ECMO due to stable angina, unstable angina, and variant angina were excluded from this study. Patients, who were clinically stable before revascularization, but received VA-ECMO for prophylactic purpose because of their poor cardiac function and high risk of expected treatment, were also excluded from the study. Finally, 255 patients were analyzed. As of the date the multidisciplinary ECMO team was founded at our institution, patients were classified into two groups: a pre-ECMO team group (before January 2014, *n* = 131) and a post-ECMO team group (after January 2014, *n* = 124). The institutional review board of Samsung Medical Center approved this study, and written informed consent was obtained.

### Multidisciplinary ECMO team

Our institution is a tertiary referral hospital with a tertiary-level intensive care unit. Since the initiation of the use of ECMO in 2004, the number of patients treated with ECMO had increased gradually. Currently, more than 100 patients are treated with ECMO each year at our institution. Cardiac surgeons or interventional cardiologists inserted VA-ECMO at bedside or in the catheterization laboratory. As far as there were no special indications, peripheral cannulation with percutaneous approach using the Seldinger technique was chosen as the initial implant method. The Capiox Emergency Bypass System (Capiox EBS™; Terumo, Inc., Tokyo, Japan) and Permanent Life Support (PLS; MAQUET, Rastatt, Germany) were used in our hospital. All patients received unfractionated heparin as an anticoagulant unless there was active bleeding. Through our hospital’s own protocol, the heparin infusion rate was adjusted to achieve the target activated clotting time of 150 to 180 s and activated partial thromboplastin time of 55 to 75 s, respectively. In the event of persistent pulmonary edema after ECMO initiation despite diuresis and inotropes, left ventricular decompression was achieved by either percutaneous atrial septostomy or surgical venting.

In January 2014, a multidisciplinary ECMO team was founded at our institution. Our ECMO team consists of interventional cardiologists, critical care physicians, cardiovascular surgeons, heart failure physicians, a pharmacist, a nutritionist, and perfusionists who were formal intensive care registered nurses and received specific ECMO training. Before the team’s establishment, attending physician, who was capable of inserting and maintaining ECMO, was responsible for running ECMO. Most of the ECMO-related decisions, from initiation to weaning, were made solely by the attending physician. In-staff training was in charge of attending physician as well. No protocol existed for maintaining ECMO. Only elective consultation to experienced cardiothoracic surgeons was possible in difficult clinical situations, with no 24-h on-call coverage by an ECMO specialist. However, after the foundation of the ECMO team, team members readily participated in the management of ECMO patients and all ECMO-related decisions, as described below. First, both the initiating and weaning of ECMO were performed under the supervision of the ECMO team. Based on our institutional ECMO protocols for indications and contraindications (Additional file 1: Table S1), the ECMO team evaluated the eligibility of the patient for ECMO and made the final decision of whether to initiate ECMO or not. The decision of weaning was also made together by the attending physician and ECMO team based on our institutional weaning criteria. Second, as part of daily rounds, echocardiography was performed to evaluate cardiac function and recovery. The pharmacist and nutritionist adjusted prescribed medications and nutritional plan in accordance with alterations of pharmacokinetics and metabolic status due to running ECMO and the critically ill status of the patient. Also, the ECMO team checked the functional status of the ECMO device including the pump, oxygenator, and cannula daily, and assessed the occurrence of ECMO-related complications and the adequacy of relevant management. Third, ECMO-trained physicians, cardiovascular surgeons, and perfusionists provided 24-h on-call coverage for ECMO patients and potential candidates. Fourth, the ECMO team was responsible for staff training. Doctors and nurses who were in charge of ECMO patients were educated by the ECMO team in order to properly manage patients according to their complicated clinical situations. Fifth, a weekly meeting was held to discuss the issues of current ECMO patients as well as review previous cases for quality assurance.

### Patient management, data collection, and study outcomes

Patient management was performed according to current standard guidelines [5, 6, 16, 17]. The choice of treatment strategy of percutaneous coronary intervention (PCI) (type, diameter, and length of stents; use of intravascular ultrasound; glycoprotein IIb/IIIa inhibitor use; and thrombus aspiration) was left to the discretion of the attending physicians. Unless there was an undisputed reason for discontinuing dual-antiplatelet therapy, all patients were recommended to take aspirin indefinitely plus a P2Y12 inhibitor for at least 1 year after the index procedure. Coronary artery bypass graft (CABG) was performed using current standard methods. The left internal mammary artery was considered preferential for revascularization of the left anterior descending artery. Patients who underwent CABG were recommended to take aspirin indefinitely. If intolerant to aspirin, taking clopidogrel as an alternative was also allowed.

Patients were prospectively registered at the time of index hospitalization. Demographic feature and cardiovascular risk factor data were collected by detailed interview with patients or their families at admission. Coronary angiographic findings and procedural history of PCI, CABG, and ECMO were gathered during hospitalization. Information about adjunctive therapies in addition to ECMO such as inotropes, mechanical ventilation, and continuous renal replacement therapy was collected at the time of discharge. Follow-up outcomes were obtained from the review of patients’ electronic medical records by research coordinators of the dedicated registry. Clinical events that occurred within a 6-month follow-up period were analyzed.

The primary outcome was in-hospital mortality. Secondary outcomes included cardiac intensive care unit (CICU) mortality, 6-month all-cause death, 6-month readmission due to heart failure, successful weaning of ECMO, complications in the CICU, length of CICU stay, duration of ECMO, duration of mechanical ventilation, and duration of continuous renal replacement therapy. All clinical outcomes were defined according to the Academic Research Consortium [18]. All deaths were considered cardiac-related unless a definite non-cardiac cause could be established. Successful weaning of ECMO was defined as maintaining hemodynamic stability after ECMO removal with or without getting durable left ventricular assist device or heart transplantation. Included complications were major bleeding, vascular complications, infection, and limb ischemia. Major bleeding was defined as bleeding in the brain, thorax, mediastinum, gastrointestinal tract, or abdomen or any fatal bleeding requiring transfusion or intervention. Vascular complications included vessel perforation, arterial dissection, and site bleeding. Site bleeding that was fatal was not included in vascular complications and included in major bleeding. Minor complications such as local hematoma were not recorded in vascular complications. Infection was defined as the presence of clinical symptoms or signs of infection with concurrent microbiological evidence of infection confirmed by blood culture during CICU stay. Limb ischemia was defined as cases requiring surgical management or having dependent performance from 0 to 2 scale on functional ambulation classification resulting from limb ischemia at discharge [19].

### Statistical analysis

Categorical variables were presented as numbers and relative frequencies and compared using the Chi-square test or Fisher’s exact test, as appropriate. Continuous variables were presented as mean ± standard deviation or median with interquartile range (Q1 to Q3) and compared using the Student’s *t* test or the Wilcoxon rank-sum test, as appropriate. The risk of in-hospital mortality was compared using logistic regression analysis and was presented as odds ratios (OR) and 95% confidence intervals (CI). To identify independent predictors of in-hospital mortality, multivariable logistic regression analysis was performed. Variables were included in the analysis if they showed a significant relation in the univariate analysis with a *p* value of less than 0.1 and were considered clinically relevant.

Cumulative incidences of clinical outcomes were calculated by Kaplan–Meier estimates and compared using a log-rank test. Cox proportional hazards regression analysis was performed to compare the risk of clinical events before and after the ECMO team establishment. Risks of clinical events were presented with hazard ratios (HR) and 95% CIs.

All probability values were two-sided and *p*-values of less than 0.05 were considered statistically significant. Statistical analyses were performed using the R Statistical Software (version 3.5.2; R Foundation for Statistical Computing, Vienna, Austria).

## Results

### Baseline and treatment characteristics

Baseline clinical and angiographic characteristics are shown in Table 1. Of the total patients, 64.3% presented with ST-segment elevation myocardial infarction (STEMI), 15.3% had out-of-hospital cardiac arrest, and 63.9% had in-hospital cardiac arrest. As for angiographic profile, the left anterior descending artery and left main coronary artery accounted for 44.7% and 23.1% of the culprit vessels, respectively. A total of 78.8% of patients presented with multivessel disease. Nevertheless, there were no differences in baseline clinical and angiographic characteristics between the two groups, except for body mass index, previous history of myocardial infarction and PCI, and baseline total bilirubin. Also, indicators of severity in ECMO patients such as ENCOURAGE score, AMI-ECMO score, and SOFA score were not different between the two groups. Regarding treatment characteristics (Table 2), successful revascularization through either PCI or CABG was higher in the post-ECMO team group than in the pre-ECMO team group (84.7% vs. 94.4%; *p* = 0.022). In STEMI patients, door-to-balloon time was shorter in the post-ECMO team group than in the pre-ECMO team group (114.0 vs. 88.0, *p* = 0.032). Extracorporeal cardiopulmonary resuscitation was performed in 67.8% of study population and there was no significant difference in proportion between the two groups. Arrest to ECMO pump-on time (for extracorporeal CPR patients only) and shock to ECMO pump-on time (for non-extracorporeal CPR patients only) were numerically shorter in the post-ECMO group than the pre-ECMO group, with no statistical significance. For supplementary treatments after ECMO insertion, the use of inotropes or vasopressors, intra-aortic balloon pump, and mechanical ventilation was significantly lower, whereas distal perfusion was more frequently performed in the post-ECMO team group than in the pre-ECMO team group.

**Table 1:** Baseline characteristics

Variables	Total (*n* = 255)	Pre-ECMO team (*n* = 131)	Post-ECMO team (*n* = 124)	*p* value
Demographics				
Age, years	64.0 ± 11.8	63.4 ± 12.0	64.7 ± 11.5	0.384
Male	200 (78.4)	105 (80.2)	95 (76.6)	0.593
Body mass index, kg/m^2^	24.4 ± 3.5	23.8 ± 3.3	25.1 ± 3.7	0.004
Cardiovascular risk factors				
Hypertension	135 (52.9)	67 (51.1)	68 (54.8)	0.642
Diabetes mellitus	128 (50.2)	67 (51.1)	61 (49.2)	0.852
Dyslipidemia	37 (14.5)	20 (15.3)	17 (13.7)	0.861
Chronic kidney disease	28 (11.0)	15 (11.5)	13 (10.5)	0.963
History of myocardial infarction	61 (23.9)	17 (13.0)	44 (35.5)	< 0.001
History of percutaneous coronary intervention	75 (29.4)	27 (20.6)	48 (38.7)	0.002
History of cerebrovascular accident	27 (10.6)	18 (13.7)	9 (7.3)	0.139
Clinical presentation				
STEMI	164 (64.3)	89 (67.9)	75 (60.5)	0.266
Systolic pressure, mmHg^a^	73.0 (67.0–82.5)	74.0 (70.0–81.0)	71.0 (67.0–83.0)	0.698
Out-of-hospital cardiac arrest	39 (15.3)	17 (13.0)	22 (17.7)	0.377
In-hospital cardiac arrest	163 (63.9)	82 (62.6)	81 (65.3)	0.747
Left ventricular ejection fraction, %	32.0 (25.0–41.0)	32.0 (25.0–45.0)	30.0 (24.5–40.0)	0.542
Laboratory findings				
Hemoglobin, g/dL	12.5 (10.4–14.7)	12.5 (10.5–14.8)	12.5 (10.2–14.6)	0.565
Prothrombin time, %	81.0 (63.0–92.0)	81.0 (63.0–91.0)	83.5 (62.0–95.5)	0.318
Total bilirubin, mg/dL	0.8 (0.5–1.1)	0.9 (0.6–1.1)	0.7 (0.4–1.0)	0.014
Creatinine, mg/dL	1.3 (1.0–1.8)	1.2 (1.0–1.8)	1.3 (1.0–1.7)	0.644
Lactic acid, mmol/L	5.5 (2.7–9.5)	6.1 (2.9–10.5)	4.7 (2.5–7.9)	0.060
Peak troponin I, ng/mL	156.5 (39.3–486.5)	153.5 (44.8–489.4)	159.6 (31.1–481.4)	0.591
Peak CK-MB, ng, mL	233.6 (60.3–423.3)	254.2 (51.5–484.2)	202.1 (62.3–383.2)	0.413
Severity score				
ENCOURAGE score	20.0 (14.0–24.5)	19.0 (14.0–24.0)	20.5 (16.0–25.0)	0.304
AMI-ECMO score	20.0 (16.0–27.0)	21.0 (16.0–27.0)	19.0 (16.0–27.0)	0.749
SOFA score	11.0 (9.0–13.0)	11.0 (9.0–13.0)	11.0 (9.0–12.0)	0.257
Angiographic findings				
Infarct-related artery				0.173
Left anterior descending artery	114 (44.7)	62 (47.3)	52 (41.9)	
Left circumflex artery	30 (11.8)	15 (11.5)	15 (12.1)	
Right coronary artery	47 (18.4)	21 (16.0)	26 (21.0)	
Left main coronary artery	59 (23.1)	28 (21.4)	31 (25.0)	
Unknown	5 (2.0)	5 (3.8)	0 (0)	
Multivessel disease	201 (78.8)	98 (74.8)	103 (83.1)	0.144

Data are presented as *n* (%), means ± standard deviations, or medians (interquartile ranges)

*AMI* acute myocardial infarction, *CK*-*MB* creatine kinase-myocardial band, *ECMO* extracorporeal membrane oxygenation, *STEMI* ST-segment elevation myocardial infarction

^a^Value measured just before ECMO insertion procedure in patients with refractory cardiogenic shock without cardiac arrest at the time of ECMO insertion

**Table 2:** Treatment characteristics

Variables	Total (*n* = 255)	Pre-ECMO team (*n* = 131)	Post-ECMO team (*n* = 124)	*p* value
Intervention				
Percutaneous coronary intervention	204 (80.0)	107 (81.7)	97 (78.2)	0.594
Coronary artery bypass graft	45 (17.6)	14 (10.7)	31 (25.0)	0.005
Door-to-balloon time, min				
STEMI	96.0 (70.0–126.0)	114.0 (73.0–156.0)	88.0 (69.0–114.5)	0.032
NSTEMI	298 (134.0–1575.5)	290.0 (120.0–758.0)	398.0 (158.0–2198.0)	0.346
Successful revascularization	228 (89.4)	111 (84.7)	117 (94.4)	0.022
Extracorporeal membrane oxygenation				
E-CPR	173 (67.8)	85 (64.9)	88 (71.0)	0.365
Arrest to ECMO pump-on time, min (E-CPR only)	35.5 (20.0–57.0)	40.0 (21.5–61.0)	34.0 (18.0–46.0)	0.147
Shock to ECMO pump-on time, min (non-E-CPR only)	108.5 (28.0–447.5)	116.5 (30.0–560.5)	105.0 (18.0–280.0)	0.325
Insertion of ECMO before revascularization	160 (62.7)	80 (61.1)	80 (64.5)	0.660
Distal perfusion	74 (29.0)	18 (13.7)	56 (45.2)	< 0.001
Cannula size, arterial, Fr	16.0 (15.0–17.0)	17.0 (16.0–17.0)	15.0 (15.0–16.5)	< 0.001
Cannula size, venous, Fr	22.0 (21.0–22.0)	21.0 (21.0–22.0)	22.0 (21.0–22.0)	< 0.001
Supplementary treatment after ECMO insertion				
Inotropes or vasopressors	243 (95.3)	129 (98.5)	114 (91.9)	0.030
Intra-aortic balloon pump	63 (24.7)	55 (42.0)	8 (6.5)	< 0.001
Mechanical ventilation	226 (88.6)	124 (94.7)	102 (82.3)	0.004
Continuous renal replacement therapy	99 (38.8)	49 (37.4)	50 (40.3)	0.727

Data are presented as *n* (%), median (interquartile range)

*ECMO* extracorporeal membrane oxygenation, *E*-*CPR* extracorporeal cardiopulmonary resuscitation, *NSTEMI* non-ST-segment elevation myocardial infarction, *STEMI* ST-segment elevation myocardial infarction

### Clinical outcomes

**Fig. 2:**
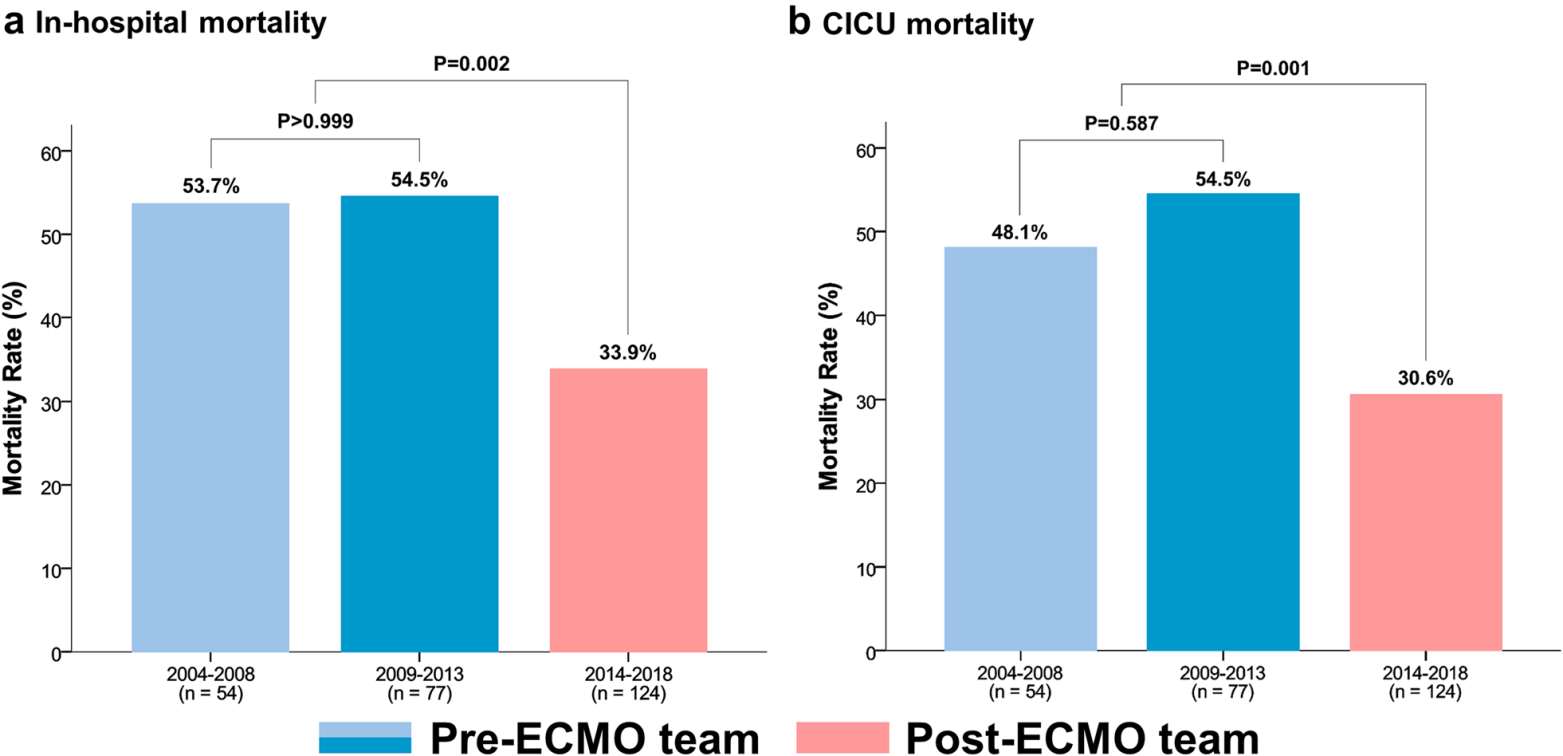
Quinquennial mortality rates between 2004 and 2018. Quinquennial (**a**) in-hospital mortality and **b** CICU mortality during the 15 years (2004 to 2018) were presented. There was no significant difference in in-hospital and CICU mortalities between subgroups (2004–2008 vs. 2009–2013) of the pre-ECMO team period. However, in-hospital and CICU mortalities were significantly lower in the post-ECMO team group than those of the pre-ECMO team group. *CICU* cardiac intensive care unit, *ECMO* extracorporeal membrane oxygenation

Clinical outcomes are presented in Table 3. In-hospital mortality occurred in 113 patients (44.3%) and CICU mortality occurred in 106 patients (41.6%). In-hospital mortality (54.2% vs. 33.9%; *p* = 0.002) and CICU mortality (51.9% vs. 30.6%; *p* = 0.001) were significantly lower in the post-ECMO team group than in the pre-ECMO team group (Fig. 2). The lower rate of in-hospital mortality in the post-ECMO team group was mainly driven by the lower rate of cardiovascular death (45.0% vs. 25.0%; *p* = 0.001). However, there were no significant differences between the two groups regarding non-cardiovascular death (9.2% vs. 8.9%; *p* > 0.99).

**Table 3:** Clinical outcomes

Variables	Total (*n* = 255)	Pre-ECMO team (*n* = 131)	Post-ECMO team (*n* = 124)	*p* value
In-hospital mortality	113 (44.3)	71 (54.2)	42 (33.9)	0.002
Cardiovascular death	90 (35.3)	59 (45.0)	31 (25.0)	0.001
Non-cardiovascular death	23 (9.0)	12 (9.2)	11 (8.9)	> 0.99
Sepsis	12 (4.7)	5 (3.8)	7 (5.6)	0.694
Bleeding	10 (3.9)	6 (4.6)	4 (3.2)	0.815
Cardiac intensive care unit mortality	106 (41.6)	68 (51.9)	38 (30.6)	0.001
Successful weaning of ECMO	169 (66.3)	75 (57.3)	94 (75.8)	0.003
Complications	102 (40.0)	66 (50.4)	36 (29.0)	0.001
Major bleeding	48 (18.8)	30 (22.9)	18 (14.5)	0.121
Vascular complications	39 (15.3)	26 (19.8)	13 (10.5)	0.057
Infection	29 (11.4)	19 (14.5)	10 (8.1)	0.155
Limb ischemia	23 (9.0)	14 (10.7)	9 (7.3)	0.461

Data are presented as *n* (%)

*ECMO* extracorporeal membrane oxygenation

**Fig. 3:**
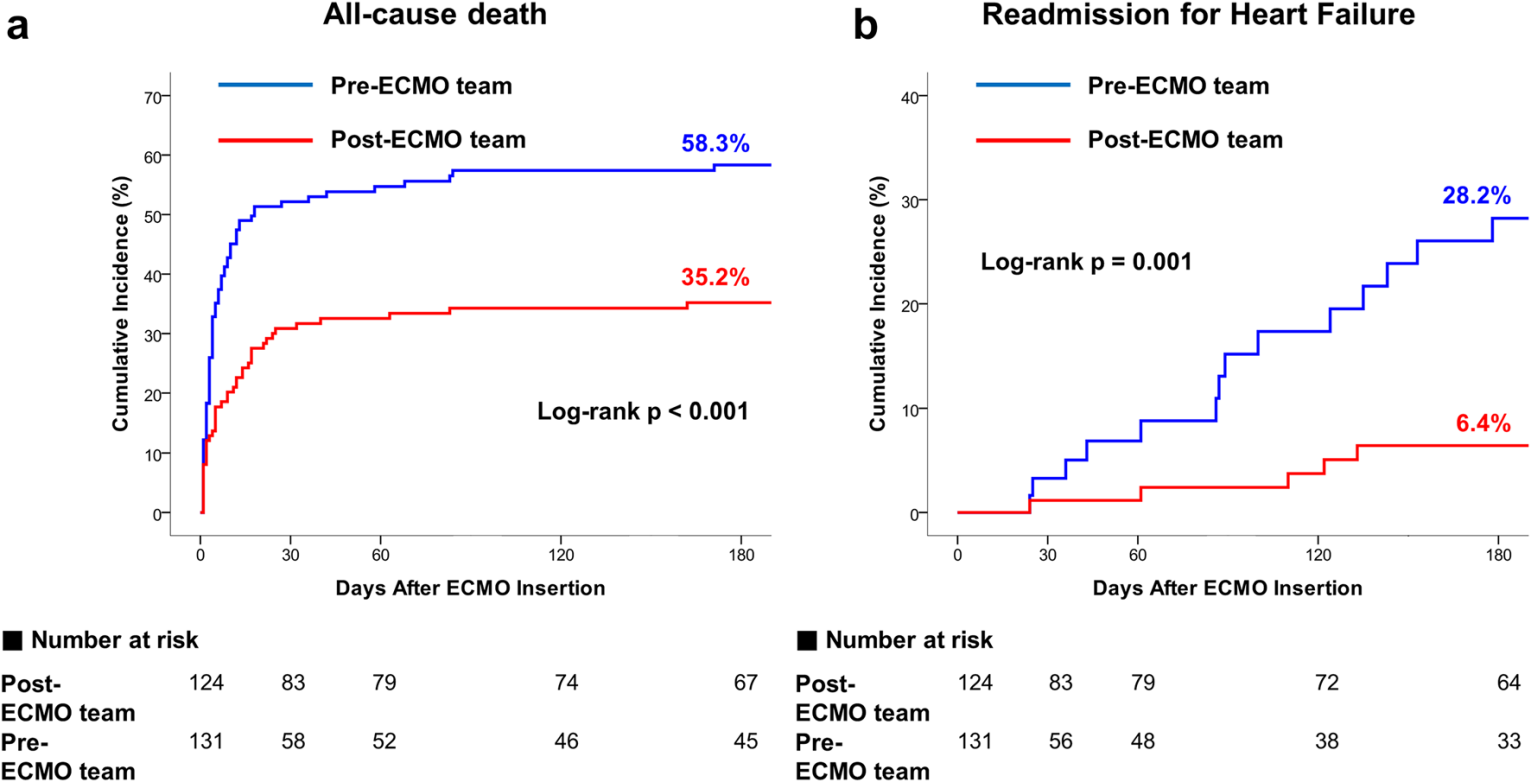
Cumulative incidence of clinical outcomes at 6 months. Kaplan–Meier curves are presented to compare the cumulative incidence of **a** all-cause death and **b** readmission for heart failure at 6 months between the pre- and post-ECMO team group. *ECMO* extracorporeal membrane oxygenation

Clinical outcomes at 6 months of follow-up showed consistent findings in relation with the primary outcome (Fig. 3). The multidisciplinary team approach was associated with significantly lower risk of all-cause death (58.3% vs. 35.2%; HR: 0.49, 95% CI 0.34–0.72; *p* < 0.001) and readmission due to heart failure (28.2% vs. 6.4%; HR: 0.21, 95% CI 0.08–0.58; *p* = 0.003) at 6 months of follow-up.

Regarding the management of VA-ECMO patients in the CICU, specific parameters are compared in Table 3 and Additional file 1: Table S2. The successful weaning of VA-ECMO (57.3% vs. 75.8%; *p* = 0.003) was higher in the post-ECMO team group than in the pre-ECMO team group. However, the length of CICU stay did not differ significantly between the two groups. Also, the duration of ECMO, mechanical ventilation, and continuous renal replacement therapy were longer in the post-ECMO team group than in the pre-ECMO team group. As for complications (i.e., major bleeding, vascular complication, infection, limb ischemia), each component tended to be lower in the post-ECMO team group than in the pre-ECMO team group, resulting in a statistically significant decrease in overall complications in the post-ECMO team group (50.4% vs. 29.0%; *p* = 0.001).

### Independent predictors of in-hospital mortality

Age, out-of-hospital cardiac arrest, successful revascularization, use of mechanical ventilation, use of continuous renal replacement therapy, annual ECMO volume and the multidisciplinary ECMO team approach showed significant relation in the univariable analysis and were included in multivariable logistic regression model (Table 4). In this model, the multidisciplinary ECMO team approach was associated with decreased risk of in-hospital mortality (adjusted OR: 0.37, 95% CI 0.20–0.67; *p* = 0.001).

**Table 4:** Predictors of in-hospital mortality

	Univariable		Multivariable	
	OR (95% CI)	*p* value	OR (95% CI)	*p* value
Multidisciplinary ECMO team approach	0.43 (0.26–0.72)	0.001	0.37 (0.20–0.67)	0.001
Age, years	1.03 (1.01–1.06)	0.003	1.05 (1.02–1.08)	< 0.001
Out-of-hospital cardiac arrest	2.97 (1.44–6.09)	0.003	5.15 (2.24–11.85)	< 0.001
Successful revascularization	0.08 (0.02–0.27)	< 0.001	0.09 (0.02–0.32)	< 0.001
Use of continuous renal replacement therapy	2.42 (1.45–4.06)	0.001	2.85 (1.59–5.12)	< 0.001
Use of mechanical ventilator	2.78 (1.14–6.76)	0.025		
Annual ECMO volume	0.96 (0.93–0.99)	0.004		

C-statistic of the logistic regression model for in-hospital mortality was 0.795 (95% CI 0.740–0.850)

Entered variables in univariate analysis for evaluating significant relation with the primary outcome included multidisciplinary approach, age, male, body mass index, hypertension, diabetes mellitus, dyslipidemia, chronic kidney disease, history of myocardial infarction, history of percutaneous coronary intervention, history of cerebrovascular accident, ST-segment elevation myocardial infarction, out-of-hospital cardiac arrest, left ventricular ejection fraction, laboratory findings in Table 1, anterior infarction, multivessel disease, percutaneous coronary intervention, coronary artery bypass graft, extracorporeal cardiopulmonary resuscitation, insertion of ECMO before revascularization, distal perfusion, use of inotropes or vasopressors, use of intra-aortic balloon pump, use of mechanical ventilation, use of continuous renal replacement therapy, overall complications and annual ECMO volume

*CI* confidence interval, *ECMO* extracorporeal membrane oxygenation, *OR* odds ratio

## Discussion

The current study is the first to evaluate the impact of a multidisciplinary ECMO team approach on clinical outcomes in AMI patients complicated by CS using data from a prospective VA-ECMO registry. The main findings were as follows. First, in-hospital mortality and CICU mortality were significantly lower in the post-ECMO team group than in the pre-ECMO team group. Second, the risks of all-cause death and readmission due to heart failure at 6-month follow-up were also significantly lower in the post-ECMO team group than in the pre-ECMO team group. Third, in a multivariable logistic regression model, multidisciplinary team approach was associated with decreased risk of in-hospital mortality in AMI patients with CS undergoing VA-ECMO.

Although multidisciplinary team approach has been recommended in the care of critically ill patients, only a few studies to date have addressed its effects on clinical outcomes [20, 21]. Also, even though the American Heart Association recommended that patients with CS be managed by a multidisciplinary team [8], nonetheless, this recommendation was primarily based on expert opinions and research regarding the association of hospital volume with clinical outcomes in CS patients, not the multidisciplinary approach [8, 22]. Furthermore, considering that inserting ECMO is a high-risk intervention and maintaining ECMO requires highly sophisticated measures, the Extracorporeal Life Support Organization guidelines recommended that ECMO be operated by multidisciplinary team including trained specialists [14]. However, there are no data about the relationship between multidisciplinary care and clinical outcomes in AMI patients complicated by CS undergoing VA-ECMO. Therefore, we aimed to investigate the impact of multidisciplinary approach in this setting and demonstrated its beneficial effect, including reduction in mortality.

Our study has several strengths. A large number of patients were observed for a sufficient follow-up period of 6 months considering that the study population was extremely severely ill patients with CS. Also, mortality as well as various treatment strategies and secondary outcomes were compared before and after the introduction of the multidisciplinary ECMO team. Lastly, the study population was extracted from a large prospective registry of a tertiary university hospital that reflects the real-world population and practices. The in-hospital mortality in our study before multidisciplinary team introduction was 54.2%, similar to that of other multicenter studies (50–60%) [4, 23]. Therefore, our study suggested that, in addition to contemporary practice of CS, the additional benefit of a multidisciplinary approach might exist.

The reasons how the multidisciplinary approach improved clinical outcomes are multifactorial in the current study. First, the multidisciplinary team consisted of experts from diverse fields. Thereby, the multidisciplinary approach enabled critically ill CS patients to receive systematic care and at the same time appropriate treatment for each problem. As a team leader, the critical care physician was closely involved and coordinated the multidisciplinary approach in order to properly manage multifaceted acute critical care [24]. Heart failure physicians were also involved in the treatment from the beginning of the initial state of shock and contributed to improve mortality not only by providing acute heart failure care, but also by maintaining the patient’s long-term cardiac function and stably directing the process toward implementing exit strategies such as ventricular assist devices and heart transplantation for indicated patients [25]. Furthermore, a pharmacist and nutritionist were included in the multidisciplinary team. The adjustment of medications according to the altered pharmacokinetics of ECMO patients led to the maintenance of drugs at the appropriate therapeutic levels without side effects [26, 27]. Likewise, customizing nutritional delivery according to the clinical status of ECMO patients helped retain an optimal nutritional status [28, 29].

Second, our institutional maintenance strategies of ECMO patients were changed in order to reduce ischemic time after multidisciplinary team implantation. If cardiopulmonary resuscitation (CPR) persisted for longer than 10 min without the return of spontaneous circulation, the ECMO team was activated and extracorporeal CPR was immediately started, unless a patient was contraindicated to receive ECMO. Also at least one primed ECMO circuit was always prepared in advance at our institution. As a result, in STEMI patients, door-to-balloon time was significantly shorter in the post-ECMO group than in the pre-ECMO group. Also arrest to ECMO pump-on time and shock to ECMO pump-on time showed shorter tendency in the post-ECMO team group than in the pre-ECMO team group.

Third, various efforts were made to reduce ECMO-related complications. During daily rounds, evaluation of cardiac function through echocardiography and modifications of clinical settings were made in order to maintain appropriate hemodynamic status. These efforts have contributed to prevent organ damage due to ischemia or overperfusion. Also, multidisciplinary team assessed the risk of ECMO-related complications by checking physical examinations and related laboratory results on a daily basis. In addition, as one of the changes in our institution’s ECMO maintenance strategies, awake ECMO was pursued unless pulmonary gas exchange was insufficient to cause upper body hypoxia. In our study, the use of mechanical ventilation was significantly lower in the post-ECMO team group and this may have played an important role in avoiding complications related to mechanical ventilation and sedation [30]. Lastly, mandatory distal perfusion, which was reported to reduce limb ischemia and even improve `survival [31], was strongly recommended. As a result, all of these diverse efforts significantly reduced the incidence of complications after the team establishment, which was considerably lower than the values shown in other studies [9].

As limitations, first, this study was an observational, prospective registry based, single-center study. Consequently, the influence of confounding bias or selection bias affecting the results of the research cannot be excluded. Although multivariable adjusted analysis was performed by adding various variables, the effects of confounding variables, such as annual ECMO volume or the learning curve of ECMO, were not completely corrected. Therefore, the results may be influenced by multifactorial causes other than multidisciplinary team. Furthermore, there might be concern about differences between the two groups when selecting patients who were appropriate candidates for using VA-ECMO. However, considering that selecting appropriate patient with team-based and protocolized decision is the effect of the multidisciplinary team, this can be considered as one of benefit of multidisciplinary team rather than the selection bias. Second, the advances in the treatment of shock patients or accumulation of experiences over time may have served as potential bias in the study. During the study period, three major randomized trials in AMI patients by CS were done [2, 32, 33]. First two studies were conducted to investigate the prognostic implications of immediate multivessel PCI and IABP, respectively, and showed no significant difference in mortality [2, 33]. On the other hand, subgroup analysis of the other study, that compared the effects of vasopressors in patients with CS, showed survival benefit of norepinephrine over dopamine [32]. These advancements seemed to have played some role in improving the clinical results. However, as shown in Fig. 2, when the patients who were treated before the multidisciplinary team establishment (2004–2013) were divided into two groups according to time, there was no significant difference in clinical outcomes between the two groups. On the other hand, there was a significant improvement in mortality between before and after 2014. Considering there was no major change in patient management other than the foundation of the multidisciplinary team, this improvement could be regarded as an additional benefit of multidisciplinary approach on the top of other advances in practice strategy or the accumulation of experiences. Third, our data could not show in detail how multidisciplinary approach affected mediating outcomes and which mediating outcomes were improved, that led to decreased mortality. This is a limitation of our retrospective study, in which data were insufficiently investigated. Further thoroughly investigated prospective study is needed to elucidate the detailed influence of multidisciplinary approach. Fourth, the multidisciplinary approach did not show a significant reduction in the duration of CICU stay and adjunctive treatment. Nonetheless, the interpretation of this result should be done with caution. This result might be related with the ability of multidisciplinary team to maintain patients stable in the long-term and save those who may have died previously. As a result, the multidisciplinary approach inevitably increased the duration of organ support.

## Conclusion

A multidisciplinary approach was associated with significantly lower in-hospital mortality in AMI patients complicated by CS who underwent VA-ECMO. Therefore, our findings support the current expert consensus that a multidisciplinary ECMO team is indispensable for improving outcomes in AMI patients with CS.

## Supplementary information

**Additional file 1: Table S1.** Indications and contraindications for VA-ECMO deployment. **Table S2.** Duration of organ support and CICU stay.

## Data Availability

Data are available from the authors upon reasonable request.

## Abbreviations

AMI: Acute myocardial infarction
CABG: Coronary artery bypass graft
CICU: Cardiac intensive care unit
CPR: Cardiopulmonary resuscitation
CS: Cardiogenic shock
ECMO: Extracorporeal membrane oxygenation
PCI: Percutaneous coronary intervention
STEMI: ST-segment elevation myocardial infarction
VA: Veno-arterial
