# Supplementary material for: Multidisciplinary team approach in acute myocardial infarction patients undergoing veno-arterial extracorporeal membrane oxygenation
Source: Ann Intensive Care. 2020 Jun 16;10:83. doi: 10.1186/s13613-020-00701-8 (PMC7296889; doi:10.1186/s13613-020-00701-8)
Supplement: Supplementary file 1 — Additional file 1: Table S1. Indications and contraindications for VA-ECMO deployment. Table S2. Duration of organ support and CICU stay. [file 13613_2020_701_MOESM1_ESM.docx]

**Additional file 1**

**Multidisciplinary team approach in acute myocardial infarction patients undergoing veno-arterial extracorporeal membrane oxygenation**

David Hong, MD^1*^; Ki Hong Choi, MD^1*^; Yang Hyun Cho, MD, PhD^2^; Su Hyun Cho, RN^2^; So Jin Park, MS^3^; Darae Kim, MD, PhD^1^; Taek Kyu Park, MD, PhD^1^; Joo Myung Lee, MD, MPH, PhD^1^; Young Bin Song, MD, PhD^1^; Jin-Oh Choi, MD, PhD^1^; Joo-Yong Hahn, MD, PhD^1^; Seung-Hyuk Choi, MD, PhD^1^; Jin-Ho Choi, MD, PhD^1^; Kiick Sung, MD, PhD^2^; Hyeon-Cheol Gwon, MD, PhD^1^; Eun-Seok Jeon, MD, PhD^1^; and Jeong Hoon Yang, MD, PhD^1,4✝^

^1^Division of Cardiology, Department of Internal Medicine, Heart Vascular Stroke Institute, Samsung Medical Center, Sungkyunkwan University School of Medicine, Seoul, Republic of Korea

^2^Department of Thoracic and Cardiovascular Surgery, Samsung Medical Center, Sungkyunkwan University School of Medicine, Seoul, Republic of Korea

^3^Department of Pharmaceutical Services, Samsung Medical Center, Seoul, Republic of Korea

^4^Department of Critical Care Medicine, Samsung Medical Center, Sungkyunkwan University School of Medicine, Seoul, Republic of Korea

**Table of contents**

**(1) Additional tables**

**(1) Additional tables**

**Table S1. Indications and contraindications for VA-ECMO deployment**

| **Indications** | **Contraindications** |
| --- | --- |
| Acute heart failure causing refractory CS that is potentially reversible in the setting of | Irreversible heart disease with no plan for transplant or VAD |
|  | Prolonged CPR (60 min.) without adequate tissue perfusion |
| Ischemic heart disease | Unwitnessed cardiac arrest |
| Post-cardiotomy | Irreversible multi-organ failure |
| Myocarditis | Severe irreversible brain injury |
| Rejection after heart transplantation | Recent cerebral hemorrhage |
| Acute decompensation of chronic heart failure | Active bleeding or bleeding diathesis |
| Pulmonary thromboembolism | Disseminated malignancy |
| Refractory ventricular tachycardia | Severe aortic regurgitation |
| Peripartum cardiomyopathy |  |
| Drug intoxication |  |
| Severe hypothermia |  |
| Bridge to transplant or VAD |  |
| Cardiac arrest that is potentially reversible |  |
| Witnessed arrest |  |
| Timing of cardiac arrest is relatively inferable |  |

Abbreviations: *CPR* cardiopulmonary resuscitation, *CS* cardiogenic shock*, ECMO* extracorporeal membrane oxygenation, *VA* veno-arterial, *VAD* ventricular assist device

**Table S2. Duration of organ support and CICU stay**

| **Variables** | **Total**  **(N=255)** | **Pre-ECMO team**  **(N=131)** | **Post-ECMO team**  **(N=124)** | **P value** |
| --- | --- | --- | --- | --- |
| Duration of ECMO, days | 4.0 (2.0-6.0) | 3.0 (2.0-5.0) | 4.0 (2.0-7.0) | 0.012 |
| Survivals^1^ | 4.0 (3.0-6.0) | 3.0 (2.0-5.0) | 4.0 (3.0-7.0) | 0.059 |
| Non-survivals^1^ | 3.0 (1.0-6.8) | 3.0 (1.0-6.0) | 4.0 (2.0-9.0) | 0.196 |
| CICU length of stay, days | 10.0 (4.0-17.0) | 9.0 (4.0-15.5) | 11.0 (6.0-20.5) | 0.053 |
| Survivals^1^ | 13.0 (8.0-23.0) | 15.0 (10.0-22.0) | 12.0 (7.0-23.0) | 0.246 |
| Non-survivals^1^ | 4.0 (2.0-10.0) | 4.0 (2.0-8.5) | 5.0 (2.0-17.0) | 0.262 |
| Duration of MV, days | 7.0 (3.0-12.0) | 6.0 (3.0-10.0) | 7.5 (4.0-14.0) | 0.041 |
| Survivals^1^ | 7.5 (4.0-12.5) | 9.0 (5.0-13.0) | 8.0 (4.0-13.0) | 0.567 |
| Non-survivals^1^ | 4.0 (2.0-10.0) | 4.0 (1.0-8.0) | 5.5 (2.0-14.5) | 0.087 |
| Duration of CRRT, days | 5.5 (3.0-12.5) | 4.0 (3.0-8.0) | 8.0 (4.0-18.0) | 0.002 |
| Survivals^1^ | 7.5 (4.0-12.5) | 5.0 (4.0-9.5) | 8.0 (4.5-17.5) | 0.104 |
| Non-survivals^1^ | 4.0 (2.0-10.0) | 3.0 (2.0-7.0) | 7.0 (3.0-18.0) | 0.035 |

Data are presented as median (interquartile range).

^1^Patients were divided according to their survival in cardiac intensive care unit. The number of survivals are 149 (pre-ECMO 63, post-ECMO 86) and the number of non-survivals are 106 (pre-ECMO 68, post-ECMO 38).

Abbreviations: *CICU* cardiac intensive care unit, *CRRT* continuous renal replacement therapy, *ECMO* extracorporeal membrane oxygenation, *MV* mechanical ventilator
